# Artificial intelligence for early detection of diabetic retinopathy: A vision transformer-based approach

**DOI:** 10.1371/journal.pone.0350854

**Published:** 2026-07-27

**Authors:** Asma ElAdel, Imen Filali, Mourad Zaied

**Affiliations:** 1 Department of Computer Science, Higher Institute of Computer Science and Multimedia, University of Gabes, Gabes, Tunisia; 2 Research Team in Intelligent Machines (RTIM) Laboratory, National School of Engineers, University of Gabes, Gabes, Tunisia; 3 Department of Computer Sciences, College of Computer and Information Sciences, Princess Nourah Bint Abdulrahman University, Riyadh, Saudi Arabia; VIT Bhopal University, INDIA

## Abstract

**Background:**

Early identification of diabetic retinopathy (DR), which is a primary cause of vision impairment globally, is a crucial phasis for effective intervention and treatment. Traditional screening workflows rely on manual diagnosis by ophthalmologists, which remains the gold standard but can be time-consuming and subject to variability due to human factors. To support and enhance the screening process, artificial intelligence (AI)-based tools have shown promise in automating DR detection, particularly with recent advances in deep learning. However, medical images with long-range dependencies and spatial linkages can be challenging for CNN-based algorithms to handle.

**Methods:**

This paper proposes a Vision Transformer (ViT)-based model, specifically using a Compact Convolutional Transformer (CCT), for early automated detection of DR. The model uses self-attention techniques to improve feature extraction and classification performance; combining three main stages: the CCT tokenizer, transformer encoder, and sequence pooling. The proposed approach was trained on public datasets (EyePACS and APTOS 2019) and evaluated against state-of-the-art deep learning architectures.

**Results:**

Our experimental findings demonstrate that ViT performs among the best in the current state of the art with an overall accuracy of 97% and F1-scores above 0.95 across all DR severity levels. Our system is primarily designed for the pre-screening stage of diabetic retinopathy workflows, enabling rapid and reliable identification of potential DR cases for further clinical evaluation.

**Conclusion:**

These results highlight the potential of transformer-based designs in medical picture analysis, as well as the implications for telemedicine and e-health solutions in real-time, especially in cases of low-resource settings.

## 1 Introduction

Diabetes is a systemic metabolic disorder associated with various vascular complications throughout the body. When diabetes coexists with other general health problems, the risk of ocular complications increases. This disease damages the small blood vessels in the retina, causing what is known as Diabetic Retinopathy (DR). Without appropriate treatment, this common complication can cause progressive damage to the blood vessels, impairing retinal function; which can lead to fluid leakage and even blockage of the blood vessels, resulting in severe vision loss or even blindness [1].

To standardize diabetic retinopathy (DR) diagnosis and management, international clinical guidelines have been established. The International Clinical Diabetic Retinopathy (ICDR) classification is one of the most widely adopted frameworks, dividing DR into five severity levels: no diabetic retinopathy (No DR), mild non-proliferative diabetic retinopathy (Mild NPDR), moderate non-proliferative diabetic retinopathy (Moderate NPDR), severe non-proliferative diabetic retinopathy (Severe NPDR), and proliferative diabetic retinopathy (PDR). This standardized staging guides treatment decisions and referral priorities in clinical practice. Early DR, according to ICDR guidelines, refers to the mild and moderate non-proliferative diabetic retinopathy levels. These stages involve the presence of microaneurysms, small hemorrhages, and limited exudates, which represent early vascular abnormalities detectable via fundus imaging. Our model specifically targets these early DR indicators, focusing on vascular changes rather than neurodegeneration or detailed angiogenesis markers. In the DR screening process, three complementary stages are typically involved: grading, triage, and pre-screening. Grading assigns a specific disease severity level based on standardized criteria, such as the ICDR five-class system. Triage involves prioritizing patients based on urgency, ensuring that high-risk cases receive prompt specialist referral while low-risk cases are monitored routinely. Pre-screening serves as the initial filtering step, automatically analyzing retinal images to determine the likelihood of DR and categorizing cases by severity to streamline subsequent triage and grading. There are several challenges in identifying and treating DR. First, analyzing retinal images requires high precision and specialized expertise, which can lead to variability and subjectivity in diagnoses. Second, traditional screening methods, while effective, are not always accessible to all populations, particularly in remote or underdeveloped areas. The need for regular monitoring and limited access to specialized care increases the risk of missed or late detection of this pathology, which can lead to serious and irreversible complications [2,3].

One promising solution to these issues is the integration of artificial intelligence (AI) into medical imaging. Deep learning, especially Convolutional Neural Networks (CNNs), has significantly improved automated DR detection by learning hierarchical image representations [4–6].

However, CNNs have limitations in capturing global dependencies and long-range spatial relationships, which are critical for precise interpretation of medical images [7]. Vision Transformers (ViTs), which use self-attention mechanisms to effectively model global dependencies, have recently become a potent substitute for CNNs. ViTs interpret images as a series of patches, allowing for a more comprehensive representation of characteristics than CNNs, which rely on local receptive fields [8].

To address these challenges, we propose a Vision Transformer (ViT)-based model specifically designed for the automated classification of DR severity levels according to the ICDR guidelines. Our approach leverages the Compact Convolutional Transformer (CCT) architecture, integrating a convolutional tokenizer, transformer encoder, and sequence pooling mechanism to extract both local and global retinal features. Additionally, our model is designed to address these early screening stages. It performs automated grading of fundus images into five ICDR-defined severity levels. This graded output serves multiple purposes: it assists pre-screening by filtering out non-DR or mild cases, and it supports triage by prioritizing moderate and severe cases for urgent specialist referral. This functionality is particularly beneficial in telemedicine settings and large-scale screening programs where resource constraints limit access to ophthalmologists.

The rest of this paper is organized as follows. Section 2 reviews existing AI-based approaches for DR detection. Section 3 presents our proposed methodology, including data set pre-processing, model architecture, and evaluation metrics. Section 4 presents the experimental results and compares the ViTs with CNNs. Then, a discussion of the proposed model is given in section 5. Finally, section 6 concludes the study and outlines future research directions.

## 2 Related work

The use of Artificial Intelligence (AI) in medical image processing has advanced significantly in recent years, particularly in the identification of diabetic retinopathy (DR). This section reviews existing approaches to medical image classification, such as classical machine learning methods, deep learning-based techniques, and the introduction of Vision Transformers (ViTs).

### 2.1 Traditional machine learning approaches

Earlier efforts to automate DR detection used a combination of manually extracted features and classical ML classifiers like Random Forests and Support Vector Machines (SVM) [9]. These technologies necessitate expert-designed features such as blood vessel segmentation, exudate detection, and microaneurysm detection [10,11]. However, these approaches exhibited constraints in scalability and robustness owing to their dependence on domain-specific feature engineering.

### 2.2 Deep learning-based approaches

The advent of deep learning has transformed DR diagnosis through Convolutional Neural Networks (CNNs), facilitating automated feature extraction from fundus images [2,4,12]. Modern CNN architectures, including InceptionV3 [13], ResNet-50 [14], and DenseNet 121 [15], have exhibited exceptional efficacy in diabetic retinopathy classification tasks [16]. In spite of their achievements, CNNs still have trouble detecting interdependencies in medical images across vast distances [6].

### 2.3 Vision transformers for diabetic retinopathy detection

Vision Transformers (ViTs) are a powerful alternative to CNNs that use self-attention mechanisms to represent global dependencies in images [7]. Unlike CNNs that rely on local receptive fields, ViTs process images as sequences of patches, allowing greater feature extraction [8,17,18]. Transformer-based models achieve cutting-edge performance in medical imaging tasks, including DR detection [19]. By including hierarchical feature extraction, hybrid models as Swin Transformers that combine CNNs with ViTs have further enhanced classification accuracy [8].

### 2.4 Limitations of existing approaches

Regarding advancements in deep learning and ViTs, issues persist in obtaining high generalizability across varied datasets [20]. Another crucial aspect for real-world implementation is to ensure that the model can be understood and accepted by clinicians [21]. Furthermore, there are substantial obstacles to large-scale deployment due to computational complexity and the need to annotate data [22].

## 3 Methodology

The Methodology section outlines the key steps involved in developing and evaluating our Compact Convolutional Transformer (CCT)-based model to diagnose diabetic retinopathy. The methodology includes the dataset description, clinical labeling standards, preprocessing, model architecture, training procedures, and evaluation metrics.

### 3.1 Exploration of data

The dataset used in this study consists of two publicly available fundus images, including the Kaggle EyePACS and the APTOS 2019 Blindness Detection datasets [23,24]. Both datasets consist of high-resolution color fundus images captured under various imaging conditions and include expert-labeled DR severity grades.

Based on ICDR, these datasets offer labeled retinal pictures grouped according to the severity level of diabetic retinopathy into five classes, as shown in Table 1.

**Table 1:** Classification of diabetic retinopathy stages.

Stage	Severity Level	Clinical Findings
0	No DR	No apparent retinopathy
1	Mild NPDR	Microaneurysms only.
2	Moderate NPDR	More microaneurysms, some hemorrhages, and cotton wool spots.
3	Severe DR	Many hemorrhages and cotton wool spots, IRMA present.
4	Proliferative DR	Neovascularization present.

**Fig 1:**
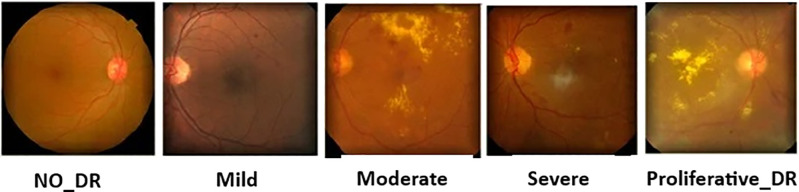
Representative fundus images showing the five severity levels of diabetic retinopathy.

The five severity levels of diabetic retinopathy are illustrated in Fig 1. Each stage exhibits distinct clinical features that guide diagnosis and treatment decisions, ranging from no apparent retinopathy to proliferative changes requiring urgent intervention.

The dataset is first analyzed for class distribution to ensure adequate representation throughout all DR stages. A summary of the data distribution is given in Table 2.

**Table 2:** Number of images per class.

Class	Number of Images
No DR	2741
Mild	740
Moderate	1898
Severe	356
Proliferate DR	590

### 3.2 Preprocessing pipeline of images

Several preparation techniques are used to enhance image quality and maximize Vision Transformer model input. Among them are noise reduction, contrast improvement with CLAHE (Contrast Limited Adaptive Histogram Equalization) [25], and normalizing.

### 3.3 Balancing of data

In order to resolve class imbalance in the dataset, oversampling of minority classes and synthetic data generation utilizing SMOTE (Synthetic Minority Over-sampling Technique) are used [26]. Furthermore, data augmentation techniques like as rotation, flipping, and brightness manipulation are used to boost dataset variability [27]. This results in a more balanced training set, preventing the model from being biased toward the majority classes.

### 3.4 Compact convolutional transformers

The Compact Convolutional Transformer (CCT) model employed in this study combines convolutional layers and transformer-based attention mechanisms to improve feature extraction and classification performance. Unlike traditional Vision Transformers (ViTs), CCT uses convolutional tokenization to better capture local spatial interactions before sending them into the transformer layers.

#### 3.4.1 Convolutional tokenization.

Standard Vision Transformers directly partition images into fixed non-overlapping patches, potentially losing fine-grained spatial details essential for detecting subtle retinal lesions (microaneurysms, small hemorrhages) in early-stage diabetic retinopathy. Our convolutional tokenizer addresses this limitation by progressively refining low-level spatial features through sequential convolutional layers before transformer encoding, rather than using fixed-sized non-overlapping patches. More specifically, this method uses three convolutional layers with trainable kernels (sizes 7×7, 3×3, 3×3) to dynamically extract token representations, resulting in a more detailed hierarchical feature from edges and textures to higher-level semantic representations.

This integrated module enables the model to capture both fine-grained retinal pathology and global structural patterns through the subsequent self-attention mechanism.

#### 3.4.2 Multi-head self-attention (MHSA).

The transformer encoder is made up of numerous layers of Multi-Head Self-Attention (MHSA), allowing the model to capture and detect long-range dependencies in the input image [28]. The MHSA mechanism is defined as follows:


$$\begin{array}{c} x_{L}=f(x_{0})\in {\mathbb{R}}^{b\times n\times d} \end{array}$$ (1)

where *x*_0_ is the input token representation, and f(·) refers to a learnable transformation applied before self-attention.

The self-attention mechanism computes attention scores using the query (*Q*), key (*K*), and value (*V*) matrices, where each is obtained via linear projections:


$$\begin{array}{c} Q=W_{Q}x_{L},\;K=W_{K}x_{L},\;V=W_{V}x_{L} \end{array}$$ (2)

where $W_{Q},W_{K},W_{V}\in {\mathbb{R}}^{d\times d_{k}}$ are learnable weight matrices, and $d_{k}$ is the key dimension. The scaled dot-product attention is calculated as:


$$\begin{array}{c} \mathrm{Attention}(Q,K,V)=\mathrm{softmax}\left(\frac{QK^{T}}{\sqrt{d_{k}}}\right)V \end{array}$$ (3)

For multi-head attention, the attention mechanism is applied independently across *h* heads:


$$\begin{array}{c} \mathrm{MHSA}(X)=\mathrm{Concat}({\text{head}}_{1},...,{\text{head}}_{h})W_{O} \end{array}$$ (4)

where $W_{O}\in {\mathbb{R}}^{hd_{k}\times d}$ is the output projection matrix.

#### 3.4.3 Positional encoding.

Since convolutional tokenization does not automatically preserve spatial order, a positional encoding technique is built into the transformer input. The positional encoding function can be determined by the following equation:


$$\begin{array}{c} x_{L}^{\prime }=\mathrm{softmax}(g(x_{L}{)}^{T})\in {\mathbb{R}}^{b\times 1\times n} \end{array}$$ (5)

$$\begin{array}{c} z=x_{L}^{\prime }⊙x_{L}=\mathrm{softmax}(g(x_{L}{)}^{T})⊙x_{L}\in {\mathbb{R}}^{b\times l\times d} \end{array}$$ (6)

where *pos* represents the token position and *i* is the embedding dimension index.

#### 3.4.4 Feed-forward network (FFN).

Each transformer block has a feed-forward network (FFN), which is made up of two fully linked layers separated by a ReLU activation function. The FFN improves feature transformation by using learnt weights and biases on the input tokens. This allows the model to capture complicated hierarchical representations of the image features.

#### 3.4.5 Classification head.

**Fig 2:**
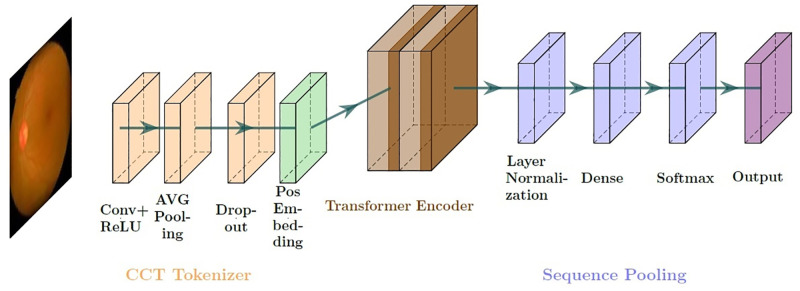
CCT-based vision transformer model architecture.

After passing through several transformer layers, the processed tokens are supplied into a Multi-Layer Perceptron (MLP) classification head. The final result is obtained using a softmax activation function, which assigns a probability distribution over the potential classes, allowing for reliable classification. Fig 2 illustrates the proposed architecture of the CCT training model.

**Fig 3:**
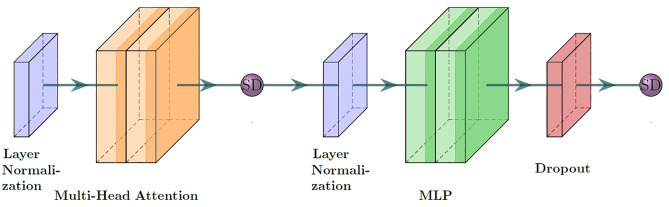
Architecture of the proposed transformer encoder.

The principal steps of the proposed Transformer Encoder are summarized in Fig 3.

### 3.5 Dataset partitioning and validation strategy

To ensure robust model evaluation and reproducibility, we employed a stratified train-test split strategy that maintains the original class distribution across both training and testing subsets. This approach is particularly critical given the inherent class imbalance in diabetic retinopathy datasets, where early-stage DR cases are significantly underrepresented compared to the “No DR” category.

- **Train-Test Split Configuration:** The combined EyePACS and APTOS 2019 datasets were partitioned using a fixed 80%–10%–10% split for training, validation, and testing, respectively. This identical partitioning strategy was applied uniformly to all models evaluated in this study, including the proposed CCTAD and all retrained baseline models (ResNet-50, EfficientNet, and DenseNet-121), ensuring a strictly fair and consistent comparison. The split was performed using stratified sampling to guarantee that each DR severity level maintains its proportional representation across all subsets. A fixed random seed (42) was used throughout all experiments to ensure full reproducibility of the split across different experimental runs.
- **CrossValidation Strategy:** During the training phase, we implemented 5-fold stratified cross-validation on the training set to optimize hyperparameters and assess model stability. In each fold, the training set was further divided into sub-training (80%) and validation (20%) sets, again maintaining class stratification.

### 3.6 Model training

**Fig 4:**
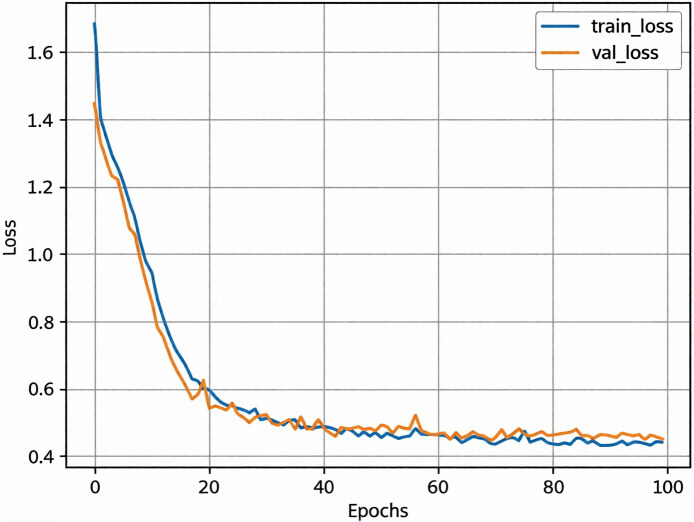
Training and validation loss curves.

The model was trained using the Adam optimizer [29] with a batch size of 32 and a learning rate of $3\times 10^{-4}$. In order to maximize accuracy in classification, the categorical cross-entropy loss function is employed. To avoid overfitting, training is done over several epochs and terminated early. See Fig 4 for the training and validation loss curves.

#### 3.6.1 Architecture configuration and hyperparameters.

To ensure full reproducibility and transparency, Table 3 provides a comprehensive specification of the CCTAD model architecture and training configuration.

**Table 3:** CCTAD model architecture and hyperparameters.

Parameter	Value
**CCT Architecture Configuration**	
Number of Transformer Layers	12
Number of Attention Heads	8
Embedding Dimension	512
FFN (Feed-Forward Network) Size	2048
Dropout Rate	0.1
Token Sequence Length	196 (14×14 patches)
Input Image Size	224×224 pixels
Convolutional Tokenizer Layers	3 (kernels: 7×7, 3×3, 3×3)
**Training Hyperparameters**	
Optimizer	Adam
Learning Rate	$3\times 10^{-4}$
Batch Size	32
Weight Decay	$1\times 10^{-5}$
Training Epochs	50 (early stopping at 47)
Loss Function	Categorical Cross-Entropy

### 3.7 Evaluation metrics

In order to evaluate the model’s efficacy, the following metrics are calculated:

- **Accuracy:** Calculates the percentage of images that are correctly labeled.
- **AUC-ROC:** Assesses the model’s ability to differentiate between various DR phases [30].
- **Sensitivity and Specificity:** Determine the model’s performance in reducing false negatives and false positives.

**Fig 5:**
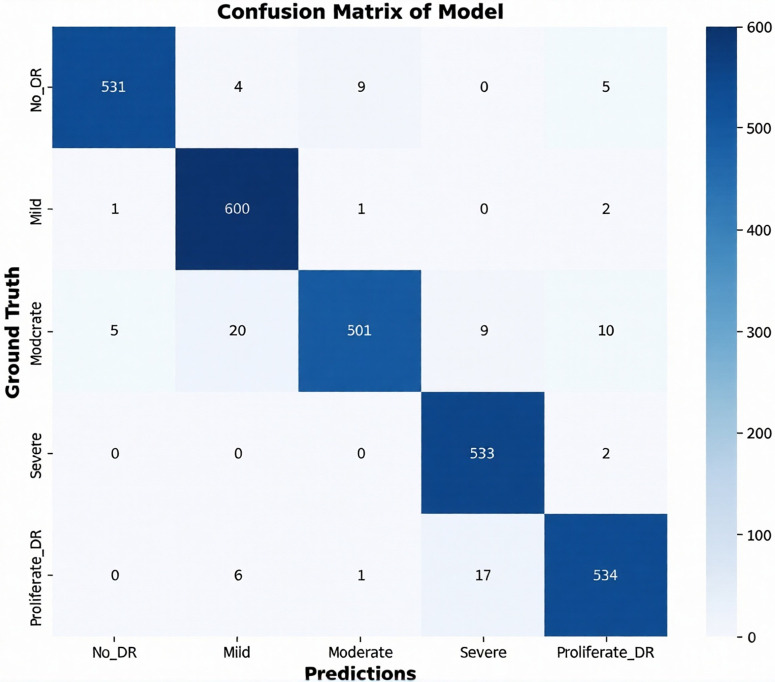
Confusion matrix of the trained model.

The confusion matrix of the trained model is shown in Fig 5.

## 4 Results

### 4.1 Computational environment

All experiments were conducted on a laptop workstation equipped with an NVIDIA GeForce RTX 4070 Laptop GPU (8 GB GDDR6 memory), AMD Ryzen 7 7435HS processor (8 cores, 16 threads, 3.10 GHz), and 32 GB DDR4 RAM. The software environment consisted of Python 3.10.12, PyTorch 2.0.1 with CUDA 11.8, and standard libraries including torchvision, scikit-learn, NumPy, and OpenCV. Training required approximately 8.5 minutes per epoch with a batch size of 32, totaling 425 minutes for 50 epochs (early stopping at epoch 47). Peak GPU memory consumption reached 6.2 GB during training, while system RAM usage peaked at 12 GB. The model achieved an average inference time of 7.3 ms per image on the test set.

These specifications make our model suitable for deployment on standard clinical workstations without requiring specialized high-end hardware, which is particularly advantageous for resource-limited healthcare settings and telemedicine applications.

### 4.2 Performance comparison between CCTAD and CNN-based models

To ensure a fair and rigorous comparison, all baseline CNN models were retrained using identical experimental conditions as the proposed CCTAD model. All models used the same 80%−10%−10% stratified split (training-validation-test) from the combined EyePACS and APTOS 2019 datasets (6,325 images total), with the same five-class ICDR severity labels (No DR, Mild, Moderate, Severe, Proliferative DR).A fixed random seed (42) was used to guarantee reproducibility of the split across all experimental runs.

To assess the effectiveness of the proposed Compact Convolutional Transformer for Automated Diagnosis (CCTAD) model in detecting diabetic retinopathy, we compare its performance to classic Convolutional Neural Network (CNN) architectures. The models are trained using the same dataset, and their performance is evaluated using common classification measures like as precision, recall, F1-score, and support for various severity levels.

Table 4 summarizes the results of the CCTAD model.

**Table 4:** Performance metrics of the CCTAD model for diabetic retinopathy classification.

Severity Level	Precision	Recall	F1-score
No DR	0.99	0.97	0.98
Mild	0.96	0.99	0.98
Moderate	0.98	0.92	0.95
Severe	0.96	1.00	0.98
Proliferate DR	0.97	0.97	0.97

As shown in Table 4, the proposed CCTAD model achieves consistently high precision and F1-scores across all DR severity levels, exceeding 0.95 in most classes. These metrics demonstrate our model’s robust discriminative ability, which is particularly critical for clinical decision-making in diabetic retinopathy screening. The model demonstrates excellent sensitivity for severe and proliferative DR (recall = 1.00 and 0.97, respectively), indicating reliable detection of advanced disease cases. This high sensitivity for advanced stages is clinically crucial, as false negatives have severe consequences: patients with undetected Severe NPDR have a 52% risk of progression to proliferative DR within one year if left untreated [31], while missed PDR cases can develop high-risk characteristics in 75% of cases within five years [32]. Our model’s performance ensures that patients requiring urgent laser photocoagulation or anti-VEGF therapy are reliably identified and referred, preventing irreversible retinal damage. A slightly lower recall (0.92) is observed for the moderate DR class, likely due to overlapping features between mild and moderate stages. However, this represents an acceptable clinical trade-off, as these patients typically require monitoring rather than immediate intervention, and regular screening protocols provide additional opportunities for detection before progression to vision-threatening stages. Overall, these results confirm the robustness and balanced performance of CCTAD across all classes.

### 4.3 Impact of preprocessing techniques

Various experimental configurations involving normalization, contrast enhancement, and noise reduction are used to evaluate the effects of preprocessing methods. The results indicate that preprocessing considerably improves model performance by reducing image variability and improving feature extraction. Contrast Limited Adaptive Histogram Equalization (CLAHE) has demonstrated a significant improvement in classification accuracy.

### 4.4 Model efficiency, interpretability, and real-world applicability

In addition to classification performance, model efficiency and interpretability are crucial for real-world clinical implementation. Table 5 compares inference time and computational cost across tested models. Despite having a substantially greater number of parameters, CCTAD achieves a reduced inference time thanks to its efficient self-attention mechanism, which allows for parallelized computing. Furthermore, attention-visualization techniques improve the interpretability of models.

**Table 5:** Comparison of computational efficiency between models.

Model	Inference Time (ms)	Parameters (Millions)
ResNet50	12.5	25.6
EfficientNet	9.8	19.3
**CCTAD (Ours)**	**7.3**	**85.2**

Table 6 compares the performance of the proposed CCTAD compared to the state-of-the-art models.

**Table 6:** Performance comparison for the APTOS dataset with the state-of-the-art models.

Model	Accuracy
CNN [12]	0.94
Inception V3 [13]	0.82
ResNet-50 [14]	0.99
VGG16 + XGBoost [15]	0.80
DenseNet 121 [15]	0.97
ViT(384_L_32) [17]	0.91
ViT-DR [18]	0.87
**CCTAD (Ours)**	**0.97**

## 5 Discussion

### 5.1 Strengths and limitations of the proposed approach

By accomplishing robust precision, recall, and F1-scores across all severity levels (As shown in section 4), the proposed Compact Convolutional Transformer for Automated Diagnosis (CCTAD) model has demonstrated high classification performance in diabetic retinopathy detection. More specifically, by integrating self-attention mechanisms, the model will able to capture both local and global dependencies within fundus images; that leading an enhancement in feature extraction stage. Furthermore, CCTAD’s efficiency in terms of inference time makes it appropriate for real-world use.

As shown in Table 6, we have compared the proposed model with recent Vision Transformer (ViT)-based approaches for diabetic retinopathy detection to better contextualize our contribution. Wu et al. [17] proposed a Swin Transformer model focused on DR grading using hierarchical attention mechanisms. While their approach effectively captures multi-level image features, it does not specifically address the challenges of early DR detection or implement class balancing techniques to diminish data skewness. Similarly, Mohan et al. [18] developed a ViT-based screening method for DR but they did not incorporate advanced preprocessing strategies or interpretability tools, such as attention visualizations, which are essential for clinical trust and transparency.

In contrast, the proposed CCTAD model offers several distinct advantages that make it particularly well-suited for real-world diabetic retinopathy screening applications:

- **Attention Mechanism for Global Feature Extraction:** Unlike CNNs that rely on local receptive fields, the Vision Transformer’s self-attention mechanism enables our model to capture long-range spatial dependencies across the entire fundus image. This is particularly advantageous for detecting subtle vascular changes distributed across multiple retinal quadrants in early-stage DR (Mild and Moderate NPDR), where microaneurysms, small hemorrhages, and exudates may appear simultaneously in different regions of the retina. The ability to model these global relationships contributes to our model’s balanced performance across all severity levels, with F1-scores exceeding 0.95 for all classes.
- **Computational Efficiency for Large-Scale Screening:** In population-level diabetic retinopathy screening programs, the bottleneck is often not solely accuracy but the combination of throughput, reliability, and interpretability. Our model’s inference time of 7.3 ms per image enables processing of approximately 137 images per second, allowing a single workstation to screen thousands of patients daily. This represents a significant advantage over ResNet-50 (12.5 ms per image) and is critical for deployment in resource-limited settings and telemedicine platforms where access to ophthalmologists is constrained.
- **Balanced Multi-Class Performance:** Our model demonstrates exceptional sensitivity for advanced DR stages that require urgent intervention (Severe NPDR: 1.00, PDR: 0.97), which is clinically crucial for preventing irreversible vision loss. This balanced performance across all severity levels, rather than optimizing for overall accuracy alone, makes CCTAD particularly suitable for pre-screening applications where reliable detection of high-risk cases is paramount.
- **Interpretability and Clinical Trust:** The attention-based architecture inherent to Vision Transformers provides opportunities for visualization of which retinal regions contribute most to classification decisions. This interpretability is essential for building clinical trust and enabling human-AI collaboration, particularly when the model flags borderline cases requiring expert review. This advantage distinguishes our approach from recent ViT-based methods that did not incorporate interpretability tools essential for clinical adoption.
- **Real-World Clinical Scenario Analysis:** In real-world screening workflows, our model’s 7.3 ms inference time enables processing approximately 137 images per second, allowing a single workstation to screen thousands of patients daily—far exceeding manual grading speeds of 2–3 minutes per patient. Combined with high sensitivity for advanced DR stages requiring urgent referral and modest hardware requirements, CCTAD is particularly suited for population-level screening in resource-limited settings and telemedicine applications where rapid triage, computational accessibility, and near-real-time feedback are critical for addressing the global burden of diabetic retinopathy.

### 5.2 Potential improvements

Several enhancements can be investigated to better improve CCTAD’s performance and usefulness:

- **Advanced Data Augmentation:** While basic augmentation techniques such as rotation and flipping are applied, more advanced methods like generative adversarial networks (GANs) and synthetic image generation can be explored to enrich the dataset and improve model robustness.
- **Hybrid Models:** Combining CCTAD with traditional CNN architectures or other deep learning models can leverage complementary strengths, potentially improving classification accuracy and reducing computational costs.
- **Real-Time Applications:** Optimization techniques such as model pruning, quantization, and knowledge distillation can be employed to reduce the computational overhead, enabling real-time inference on mobile and embedded devices.

### 5.3 Ethical considerations in AI-based medical diagnostics

As AI-driven models are increasingly adopted in healthcare, ethical considerations must be addressed to ensure responsible deployment. Key concerns include:

- **Bias and Fairness:** AI models trained on biased datasets may exhibit disparities in performance across different demographic groups. Ensuring diverse and representative datasets is crucial for equitable healthcare outcomes.
- **Transparency and Interpretability:** Clinicians require a clear understanding of AI model decisions. Attention visualization and explainable AI (XAI) techniques should be integrated to provide insights into the decision-making process.
- **Data Privacy and Security:** Patient data must be handled with strict privacy regulations. Secure data-sharing protocols and federated learning approaches can help maintain confidentiality while enabling collaborative model training.

Addressing these challenges will contribute to the safe and ethical deployment of AI-based diagnostic systems, ultimately improving patient outcomes and trust in automated healthcare solutions.

## 6 Conclusion and future work

### 6.1 Summary of key findings

The key findings in this study can be presented as follow:

- A model for DR identification called Compact Convolutional Transformer for Automated Diagnosis (CCTAD) is proposed in this study.
- An improvement of feature extraction, by integrating convolutional tokenization with self-attention mechanisms, that effectively capturing both local and global image features.
- According to obtained results, we can ensure that the proposed CCTAD have achieved high precision, recall, and F1-scores across different severity levels, outperforming conventional CNN-based models in terms of classification accuracy and efficiency.
- Additionally, preprocessing techniques such as contrast enhancement and noise reduction significantly contributed and greatly helped to enhance model performance.

### 6.2 Future research directions

To further improve CC TAD’s functionality and applicability, future studies will concentrate on interesting directions as optimizing computational efficiency, integrating the system into real-world deployment in healthcare applications.

- **Model Optimization:**Various techniques, like Pruning, quantization, and knowledge distillation, can be used as methods of reducing model complexity and enabling deployment on resource-constrained devices.
- **Clinical Validation:** The generalizability and reliability of CCTAD in real healthcare settings will be evaluated through extensive validation on varied clinical datasets and real-world patient data.
- **Real-world feature:** In real-world screening programs, a significant portion of fundus images may be ungradable due to blur, low illumination, or motion artifacts. Although the datasets used in this study predominantly contained high-quality, expert-verified images, future implementations of the proposed CCTAD system will integrate an automated image-quality assessment module. This component will use sharpness metrics (e.g., variance of Laplacian), contrast evaluation, and deep-learning–based IQA networks to identify and exclude ungradable images before analysis. Incorporating this module will enhance the reliability and safety of automated DR screening in large-scale or teleophthalmology settings.
- **Deployment in Healthcare Settings:**Ensure that AI systems and medical professionals can work together seamlessly by investigating the integration of CCTAD into automated screening systems and telemedicine platforms.

## Supporting information

S1 File APTOS dataset link. (TXT)

S2 File EyePACS dataset. (TXT)
