# Correction: Artificial intelligence for early detection of diabetic retinopathy: A vision transformer-based approach

**DOI:** 10.1371/journal.pone.0357114

**Published:** 2026-08-26

**Authors:** The PLOS One Staff

There is an error in affiliation 3 for author Imen Filali. The correct affiliation 3 is: Department of Computer Sciences, College of Computer and Information Sciences, Princess Nourah bint Abdulrahman University, P.O. Box 84428, Riyadh 11671, Saudi Arabia.

The publisher apologizes for the error.
